# Evaluation of Dietary and Alcohol Drinking Patterns in Patients with Excess Body Weight in a Spanish Cohort: Impact on Cardiometabolic Risk Factors

**DOI:** 10.3390/nu15224824

**Published:** 2023-11-17

**Authors:** Maite Aguas-Ayesa, Patricia Yárnoz-Esquiroz, Laura Olazarán, Carolina M. Perdomo, Marta García-Goñi, Patricia Andrada, Javier Escalada, Camilo Silva, Ascensión Marcos, Gema Frühbeck

**Affiliations:** 1 Department of Endocrinology & Nutrition, Clínica Universidad de Navarra, 31008 Pamplona, Spain; 2 CIBER Fisiopatología de la Obesidad y Nutrición (CIBEROBN), Instituto de Salud Carlos III, 31008 Pamplona, Spain; 3 Obesity and Adipobiology Group, Instituto de Investigación Sanitaria de Navarra (IdiSNA), 31008 Pamplona, Spain; 4 Immunonutrition Research Group, Department of Metabolism and Nutrition, Institute of Food Science and Technology and Nutrition (ICTAN)—CSIC, 28040 Madrid, Spain

**Keywords:** overweight, obesity, dietary pattern, alcoholic beverages, beer, adiposity, gender differences, cardiometabolic risk factors

## Abstract

Unhealthy dietary habits and sedentarism coexist with a rising incidence of excess weight and associated comorbidities. We aimed to analyze the dietary and drinking patterns of patients with excess weight, their main characteristics, plausible gender differences and impact on cardiometabolic risk factors, with a particular focus on the potential contribution of beer consumption. Data from 200 consecutive volunteers (38 ± 12 years; 72% females) living with overweight or class I obesity attending the obesity unit to lose weight were studied. Food frequency questionnaires and 24 h recalls were used. Reduced-rank regression (RRR) analysis was applied to identify dietary patterns (DPs). Anthropometry, total and visceral fat, indirect calorimetry, physical activity level, comorbidities and circulating cardiometabolic risk factors were assessed. Study participants showed high waist circumference, adiposity, insulin resistance, dyslipidemia, pro-inflammatory adipokines and low anti-inflammatory factors like adiponectin and interleukin-4. A low-fiber, high-fat, energy-dense DP was observed. BMI showed a statistically significant (*p* < 0.05) correlation with energy density (r = 0.80) as well as percentage of energy derived from fat (r = 0.61). Excess weight was associated with a DP low in vegetables, legumes and whole grains at the same time as being high in sweets, sugar-sweetened beverages, fat spreads, and processed meats. RRR analysis identified a DP characterized by high energy density and saturated fat exhibiting negative loadings (>−0.30) for green leafy vegetables, legumes, and fruits at the same time as showing positive factor loadings (>0.30) for processed foods, fat spreads, sugar-sweetened beverages, and sweets. Interestingly, for both women and men, wine represented globally the main source of total alcohol intake (*p* < 0.05) as compared to beer and distillates. Beer consumption cannot be blamed as the main culprit of excess weight. Capturing the DP provides more clinically relevant and useful information. The focus on consumption of single nutrients does not resemble real-world intake behaviors.

## 1. Introduction

A dramatic increase in obesity prevalence has been witnessed in the last decades [1,2,3,4,5]. The World Health Organization (WHO) recognizes that currently 39% of the world’s population presents overweight, while 13% is living with obesity [6]. Similar demographic trends are observed in Spain, with an estimated prevalence of overweight of 39.3% and obesity of 21.6% in Spanish adults aged 25–64 years [7,8]. Noteworthily, excess weight is an independent risk factor for type 2 diabetes (T2D), cardiovascular diseases (CVD), hypertension, non-alcoholic fatty liver, dyslipidemia, and certain cancers, among others [9,10]. Thus, excess weight and its accompanying comorbidities embody one of the main health problems worldwide [11,12].

Unhealthy dietary habits and sedentarism coexist with the rising incidence of overweight, obesity, the associated comorbidities and chronic disease [13,14,15]. After decades of proposed policies, obesity prevalence has not been successfully decreased. Despite the knowledge of obesity as a chronic, treatable disease, its management still faces challenges regarding the most adequate treatment approach [16]. In this context, nutrition, among others, is a key element in health and disease with a maintained caloric imbalance between energy expenditure and intake over time promoting the development of obesity [17,18]. The diverse components that influence total energy expenditure (TEE) include thermic effect of food (TEF), resting energy expenditure (REE) and physical activity [19,20]. While sophisticated techniques like doubly labelled water or direct calorimetry can be applied to measure TEE, they are mainly being carried out for research purposes [21,22]. In the clinical setting, however, REE can be determined by indirect calorimetry, which combined with physical activity level (PAL) assessment can inform about TEE [23].

Dietary interventions systematically offered during decades to lose weight have lacked patient experience, thereby being vastly ineffective in the long term. Lifestyle choices and in particular dietary changes are often seen as something that people living with obesity (PlwO) should improve by themselves, representing one of the lacunae in management. Traditionally, emphasis has been placed on avoiding particularly detrimental foods. Overconsumption of energy based on nutrient-poor, energy-dense foods and drinks reportedly elevates the risk of excess body weight and also increases the risk of obesity comorbidities [24]. Noteworthily, these dietary and drinking patterns rely on regional and cultural beliefs, as do some unsupervised weight-loss attempts. In Spain, people attempting to lose weight usually eliminate bread and beer from their diets, since these food items are popularly considered as the main culprits of excess weight. However, this belief has not been supported by scientific evidence specifically addressing this question.

In this context, we hypothesized that the excess energy intake cannot be blamed on a single food item like beer. Therefore, the rationale of the present study relied on the fact that capturing the multiple dietary options comprising a dietary pattern (DP) is key for better understanding energy imbalance in excess weight. To that end, we aimed to analyze the dietary and drinking patterns of patients with excess weight to decipher their main characteristics and the potential existence of gender differences and their impact on cardiometabolic risk factors, with a particular focus on the potential contribution of beer consumption. This information will enable the possibility of establishing more evidence-based dietary recommendations based on actual DPs to achieve a more holistic body weight regulation rather than condemning single foods.

## 2. Materials and Methods

### 2.1. Patient Selection and Study Design

This one-arm, open-label, exploratory, cross-sectional study collected momentary data from 200 consecutive male and female White patients with overweight or class I obesity attending the Endocrinology and Nutrition Department of the University of Navarra Clinic to lose weight. Criteria for inclusion consisted of being of either gender, a non-smoker, aged between 18–65 years, having a body mass index (BMI) between 25.0–35.0 kg/m^2^, not taking medication, and having no psychiatric pathology. All subjects involved in the study provided informed consent. Criteria for exclusion included other severe systemic diseases unrelated to excess weight, infections or inflammatory processes, cancer, or marked nephro- or hepatopathy, other than metabolic-associated fatty liver disease, pregnancy or lactation, being vegetarian, vegan, or persons with special dietary requirements. The sample population thus represents a homogeneous study group comprised of the habitual patients attending our department and clinic who share a common ethnic, cultural and socioeconomic background. In this line, neither physical activity nor lifestyle factors accounted as potential confounders due to the shared sedentarism and daily living style habits. This is a substudy of the trial registered with ClinicalTrials.gov, number NCT01055626. The study design was accepted from a scientific and ethical point of view by the Ethical Committee of the Hospital, receiving approval number 30/2006 further updated in number 2020.041. All procedures conformed to the guidelines of the Declaration of Helsinki as well as its subsequent revisions; the research protocol was approved by the Universidad de Navarra’s Ethical Committee (protocol 2020.041).

### 2.2. Anthropometry, Body Composition and Resting Energy Expenditure Determination

To obtain the BMI, weight was measured in kg with a 0.1 kg precision and height was determined in meters with a Harpenden stadiometer (Holtain Ltd., Crymych, UK). To measure waist circumference (WC), a non-elastic tape was placed on the midaxillary line at the midpoint between the rib cage and the iliac crest. In order to ensure uniformity in anthropometric measurements and to limit interobserver variability, anthropometric measurements were obtained by the same researcher with the ISAK (International Society for the Advancement of Kinanthropometry) certification. Total percent body fat was determined using the Bod-Pod^®^, based on air-displacement plethysmography (Life Measurements, Concord, CA, USA) applying the equation of Siri, as described before [25]. Air-displacement plethysmography reportedly closely agrees with hydrodensitometry, or underwater weighing, traditionally considered the gold standard [26]. Trunk fat and visceral adiposity were quantified by abdominal bioimpedance using a Tanita AB-140 device (Tanita Corp., Tokyo, Japan), as previously reported [27]. Trunk fat includes both subcutaneous and intra-abdominal adipose tissue, while visceral fat is expressed in arbitrary units, which, as indicated by the manufacturer, multiplied by 10 equates to the visceral adiposity area assessed by computed tomography expressed in cm^2^. Blood pressure was determined in a semi-sitting position with a sphygmomanometer at the non-dominant arm at at least 3 different time intervals to obtain the mean following a 5 min rest. After a 12 h fasting period, production of carbon dioxide and consumed oxygen were measured at thermoneutrality by indirect calorimetry (Vmax29, SensorMedics Corporation, Yorba Linda, CA, USA) to determine REE [23]. A validated questionnaire [28] was applied to estimate the physical activity level (PAL) to obtain the TEE of all study participants.

### 2.3. Dietetic History and Nutritional Information

Information on dietary intake was collected by trained dietitians using 24 h recall records together with a standardized dietary intake toolkit to help with quantification. Interviews covered both workdays and weekends to ensure a proportional representation. All types of drinks (including alcoholic beverages) and foods were registered, calculating the size of portions with habitual brands, household estimates of package weights, and where appropriate, also collecting ways of cooking [29]. Foods consumed in quantities over 15 g or one tablespoon were included. Mean intakes of the single macronutrients, specific food groups and individual DPs were calculated across the 24 h recalls. While 24 h recalls are more appropriate to gauge the eating patterns of a specific demography, food frequency questionnaires (FFQs) are more appropriate for tracking individual dietary patterns. Foods not consumed on a daily basis but still relevant to a person’s diet were captured via FFQs that rely on a longer recall period. Bias in FFQs was minimized by conducting dietitian-led interviews instead of self-reported questionnaires.

In order to develop the cluster analysis, the food group information needs to be obtained. The 24 h recall data identified 16 food groups, which included eggs, dairy products and milk, seafood and fish, meat, cold meats, legumes, cereals, starchy staples, leafy dark green vegetables, roots and tubers, other vegetables, fruits, nuts and seeds, oils, fats and spreads, sweets, and beverages. Mean dietary intake was obtained by the multiple-pass approach applied, including the three-steps method, namely ‘quick listing’, ‘detailed food and beverage describing’ and ‘final reviewing’, to enhance full and precise recalling to decrease respondent burden. Dietary adequacy was analyzed via individual diet diversity scores by means of summing the number of nine aggregated food groups (eggs, meats, fish, milk and dairy products, starchy staples, vegetables, fruits, legumes, nuts and seeds) [30]. The actual complexities of the general population’s eating habits can be better captured by DP analysis, as it accounts for the interactive and synergistic effects of different nutrients and food groups [31,32]. Reduced-rank regression (RRR) uses data-driven analysis of real-world eating behaviors aimed at identifying DPs in food intake reports via the combination of current knowledge and plausible mechanisms of diet-related disease [33]. Thereby, establishment of the relative relevance of a number of nutrient-related mechanisms plausibly linking diet to weight or cardiometabolic risk can be achieved by RRR [34]. The combination of nutritional information is important, since to reliably assess diet–disease relations the within-person variability in dietary consumption needs to be contemplated [35].

### 2.4. Blood Analyses

Anthropometric as well as body composition determinations were carried out on the same day together with the blood extraction. To avoid plausible confounders based on hormone rhythmicity, blood samples were taken in the morning after an overnight fast of at least 10 h. An automated analyzer (Roche, Basel, Switzerland/Hitachi, Tokyo, Japan Modular P800) was used to quantify plasma glucose [36]. An enzyme-amplified chemiluminescence assay (Immulite^®^, Diagnostic Products Corp., Los Angeles, CA, USA) was used to measure insulin concentrations, as in prior studies [37,38]. Insulin sensitivity and resistance were estimated via the quantitative insulin-sensitivity check index (QUICKI) [39] and the homeostatic model assessment (HOMA) [40], respectively [41,42]. The lipid profile was assessed via enzymatic spectrophotometric methods (Roche, Basel, Switzerland) for triglyceride and total cholesterol determination [43]. Colorimetry (Beckman Synchron^®^ CX analyzer, Beckman Instruments, Ltd., Bucks, UK) was applied to determine high-density lipoprotein (HDL-cholesterol), while low-density lipoprotein (LDL-cholesterol) was obtained from the Friedewald formula. The ultrasensitive assay (Tina-quant^®^ CRP, Roche, Basel, Switzerland) was used to measure high-sensitivity C-reactive protein (CRP). The Clauss method was applied to analyze circulating fibrinogen levels (Hemoliance^®^, Instrumentation Laboratory, Barcelona, Spain). A fluorescence polarization immunoassay (Axis Biochemicals ASA, Oslo, Norway) was used for homocysteine determination with an IMX analyzer (Abbott, Abbott Park, IL, USA). An automated analyzer (Roche/Hitachi Modular P800) analyzed aspartate aminotransferase (AST), alanine aminotransferase (ALT), γ-glutamyltransferase (γ-GT), uric acid, and creatinine [44,45].

Targeted adipokines and cytokines were selected to reflect the impact on inflammation of the DP identified. Furthermore, well-known cardiometabolic risk factors were also included to evaluate the effects of excess weight. Determination of the circulating concentrations of pro-inflammatory adipo-cytokines and interleukines (IL) included leptin, which works also as a lipostat, by quantification via a double-antibody radioimmunoassay method (Linco Research, Inc., St. Charles, MO, USA) with 5.0% and 4.5% intra-and inter-assay coefficients of variation (CV), respectively [46]. An enzyme-linked immunosorbent assay (ELISA) was applied for the determination of adiponectin, which operates as an insulin sensitizer and cardioprotective factor (BioVendor, Brno, Czech Republic) with intra-and inter-assay CVs of 6.7% and 7.8%, respectively. Chitinase-3-like protein 1 (YKL-40), osteopontin (OPN), lipocalin-2 (LCN-2) and tenascin C (TNC) are well-known proinflammatory factors and were assessed with commercially available ELISA kits of R&D systems (Minneapolis, MN, USA) and IBL International GMBH (Hamburg, Germany), which exhibited the following intra- and interassay CVs: 4.6% and 6.0% for YKL-40; 3.2 and 5.9% for OPN; 3.7% and 6.5% for LCN-2; 4.9% and 5.4% for TNC, respectively. A high-sensitivity ELISA kit was used to measure TNF-*α* (no. HSTAA00D, R&D Systems, Minneapolis, MN, USA), which also has relevant autocrine modulation, with intra- and inter-assay CVs of 5.0% and 5.7%, respectively. Since some interleukins have proinflammatory effects while others exert anti-inflammatory properties, representative cytokines of both of them were assessed. IL-1β concentrations were determined by ELISA (RAF052R, BioVendor), while IL-6 was analyzed by ELISA (DY206-05, R&D Systems) with an intra-assay CV of 5.4% and an inter-assay CV of 11.4%. Monocyte chemoattractant protein-1 (MCP-1) and IL-10 serum levels were analyzed via the Human Quantikine^®^ Immunoassay kits (R&D Systems), while IL-4 and IL-8 were determined with ELISA commercial kits (Genzyme, Cambridge, MA, USA) and all within-assay CV being less than 8%. Similarly, circulating concentrations of IL-32 were assessed with an ELISA commercial kit (CUSABIO, College Park, MD, USA) yielding intra- and interassay CVs < 8.0 and 10.0%, respectively.

### 2.5. Statistical Analyses

Mean ± standard errors of the mean (SEM) were used to present data. The Kolmogorov–Smirnov test assessed the normal distribution while Levene’s test analyzed homogeneity of variance. Student’s *t* tests were applied for comparison of two groups. ANOVA and χ^2^ analysis, as pertinent, were carried out to analyze gender distribution differences, type of alcoholic beverage and frequency distribution with categorical variables. Pearson’s correlation coefficients (*r*) interrogated the correlations between anthropometric measures, energy density and source. SPSS version 23 (SPSS, Chicago, IL, USA) was used for all calculations. Because of the homogeneity of the study sample, the possibility of potential confounders due to differences in race, ethnicity, lifestyle factors, educational level, and socioeconomic background, among others, was not observed. Statistical significance was established at 0.05.

The mean intakes of specific nutrients, individual food groups and DPs were analyzed across the 24 h dietary assessment. Additionally to comparing reproducibility estimates for consumption of food groups determined by the 24 h assessment as well as the FFQ, the comparison with the rankings of usual food group intake was established. DPs were derived from RRR in order to establish the linear function of food groups (as predictor variables) via maximization of the explained response variation [34]. Different DPs were derived from each analysis, encompassing the whole spectrum from the ‘more healthy’ to the ‘less healthy’ profile. No notable differences between the patterns derived from percentage energy, adjusting weight for intake of total energy or directly using weight values, were observed [47]. DP scores were considered in categorical and continuous forms applying the Statistical Analysis System software version 9.4 (SAS Institute, Cary, NC, USA). Analyses of weighted Pearson correlation evaluated the correlations between DP scores and their subcomponents. DP z-scores were calculated for each participant by the RRR model as a linear, weighted combination of all of their standardized food group intakes with unique weights for each DP. Individual z-scores for each participant were obtained for each DP derived, whereby a higher z-score corresponds to a higher adherence to the DP identified. Thus, intake of foods with a negative factor loading decreased the DP z-score, while consumption of foods with a positive factor loading increased the DP z-score. Factor loadings  ≤ −0.20 and ≥ 0.20 for food groups were significant and taken as the more prominent negative and positive contributors to the DP z-scores, respectively.

## 3. Results

### 3.1. Study Cohort Characteristics

The first 200 participants attending the Endocrinology and Nutrition Department for weight loss exhibited a mean age of 38 ± 12 years with a majority of females (72%). This finding is in agreement with males and females exhibiting different behaviors and attitudes as regards weight perception, with males being less likely to perceive themselves as overweight than females [48]. Moreover, this misperception translates into males being less likely than females to seek advice for weight loss [49]. The anthropometric and clinical characteristics of study participants are summarized in Table 1. Participants belonged to both the overweight and obesity categories, exhibiting a mean BMI within class I obesity accompanied by a high WC together with an elevated total and visceral adiposity. Although the mean baseline glycemia was within the normal range, the high HOMA index evidenced that it was achieved at the expense of a high insulin resistance accompanied by a low insulin sensitivity, as indicated by the QUICKI index. Participants had a normal hepatic and renal function. Mean blood pressure, both systolic and diastolic, were within the normal range. As expected with the high adiposity of the participants, metabolic alterations consistent in dyslipidemia, elevated proinflammatory adipokines like leptin, osteopontin, lipocalin 2, tenascin C, YKL-40, TNF-α, MCP-1, IL-1β, and IL-32, as well as high circulating concentrations of cardiometabolic risk factors like CRP, von Willebrandt factor, homocysteine and fibrinogen together with low anti-inflammatory adipokines like adiponectin and cytokines such as interleukin-4, were observed.

### 3.2. Energy Imbalance, Dietary and Drinking Patterns

Globally, participants exhibited an excess energy intake over expenditure. Furthermore, gender-specific differences were observed, with men showing a significantly higher (*p* < 0.05) intake of calories derived from alcohol than women. However, energy intake derived from alcohol was not high (2.9 and 4.5% of total energy intake for females and males, respectively) showing that this was not the main factor explaining the energy imbalance between intake and expenditure (Table 2). This was further corroborated, as 5% of the participants attending the Department for weight loss were nondrinkers. The minimal and maximal values show the wide range in intake of both females and males.

Interestingly, for both women and men, wine represented globally the main source of total alcohol intake (*p* < 0.05), as compared to beer and distillates (Table 3). This pattern was maintained in female participants that had either less than one drink per week or 5–7 drinks per week, whereas in males having less than 1 drink per week beer was the main type of alcoholic beverage. Data in low- and high-frequency consumption provide clinically interesting information. As expected, the proportion of males consuming beer was significantly higher (*p* < 0.05) than that of women in global terms, while females consumed significantly more (*p* < 0.05) wine than men without significant differences between sexes regarding liquors and distilled spirits.

**Figure 1:**
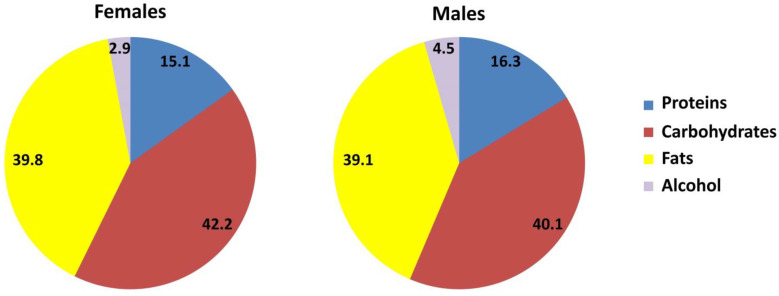
Percentage distribution of macronutrients of the total energy intake. Mean values expressed as percentage (%) of total energy intake.

Figure 1 shows the macronutrient distribution derived from the total energy intake for both males and females obtained during the dietetic history. Both genders exhibited an inadequate intake resulting from a high intake of fats and a low intake of complex carbohydrates as compared to healthy dietary recommendations.

**Figure 2:**
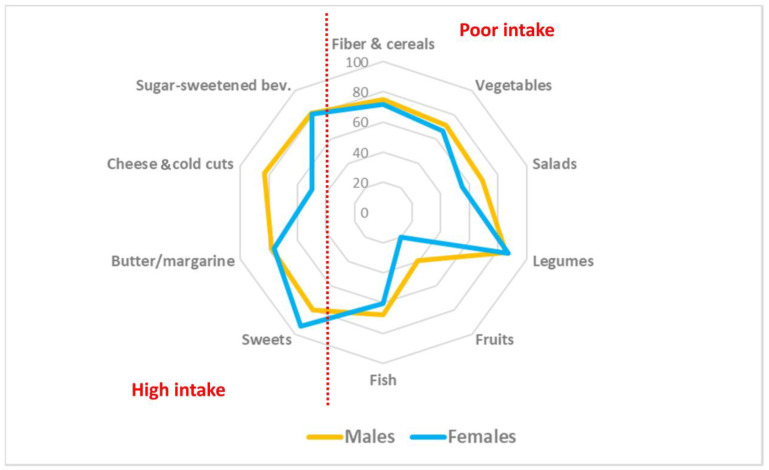
Radial plot illustrating the differences between males and females in the intake of some of the main elements characterizing dietary pattern. The different radial lines (0–100) indicate the percentage of study participants exhibiting on the right side of the graph a poor intake of fibers and cereals, vegetables, salads, legumes, fruits and fish, whereas on the left side the graph reflects the percentage of study participants showing a high intake of sugar-sweetened beverages, cheese, butter and fat spreads, and sweets.

**Figure 3:**
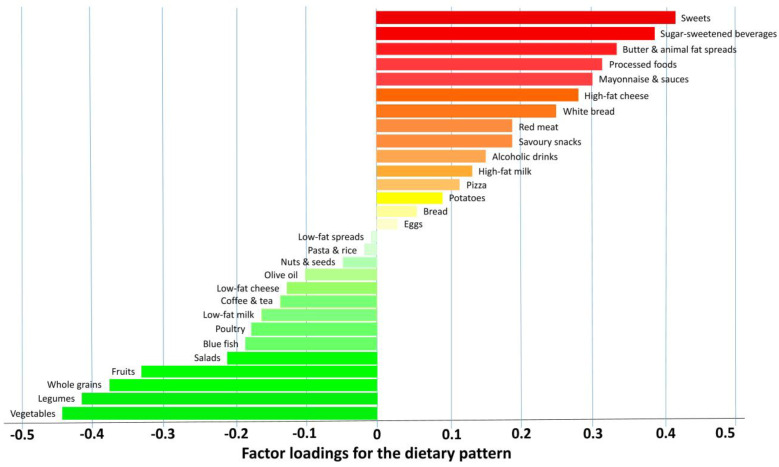
Dietary pattern factor loadings derived from reduced-rank regression.

The intake of sugar-sweetened beverages was above the optimal intake for both males and females. In addition, the energy consumption of healthy foods was suboptimal in the cohort studied (Table 4 and Table 5). Noteworthily, frequency of intake of specific foods in relation to the frequency of consumption of alcoholic drinks per week yields valuable information as regards the impact of healthy and unhealthy food groups. Men exhibited generally a higher consumption of both unhealthy and healthy foods than women. Overweight and obesity were both associated with a DP low in vegetables, legumes and whole grains at the same time as being high in sweets, sugar-sweetened beverages, spreads, and processed meats (Figure 2). RRR analysis identified a DP characterized by high energy density and saturated fat exhibiting negative loadings (>−0.30) for green leafy vegetables, legumes, and fruits at the same time as showing positive factor loadings (>0.30) for processed foods, fat spreads, sugar-sweetened beverages, and sweets (Figure 3). A low-fiber, high-fat, energy-dense DP was observed in the study participants. BMI showed a statistically significant (*p* < 0.05) correlation with energy density (r = 0.80) as well as percent of energy derived from fat (r = 0.61), while positive high factor loadings significantly (*p* < 0.05) related to adverse biomarkers profiles.

## 4. Discussion

The present study provides evidence about the anthropometric and cardiometabolic profile of patients with excess weight of this cohort. The detailed nutritional information gathered within the experimental design combining 24 h recalls, FFQs and RRR analysis allows the detailed description of their quantitative and qualitative characteristics. Macronutrient analysis highlighted the high energy intake coming from fats at the expense of a low consumption of complex carbohydrates. Noteworthily, excess weight was associated with a DP low in vegetables, legumes, whole grains and fruits at the same time as being high in sweets, sugar-sweetened beverages, fat spreads, and processed foods. RRR analysis identified a DP characterized by high energy density and saturated fat exhibiting negative loadings (>−0.30) for green leafy vegetables, legumes, and fruits at the same time as showing positive factor loadings (>0.30) for processed foods, fat spreads, sugar-sweetened beverages, and sweets. A high intake of sugar-sweetened beverages was shown in both males and females. Interestingly, high intakes of low-fiber bread, confectionery, chocolate, butter and added sugars, together with low intakes of vegetables and fresh fruits, conform to DPs that have been associated with a higher T2D incidence, in particular in PlwO and among younger people [24]. Similarly, an obesity-related DP has been prospectively associated with an elevated risk of CVD, an increased incidence of site-specific and overall cancers as well as all-cause mortality [50,51,52]. Likewise, higher consumption of ultra-processed food (UPF) in prospective cohort studies reportedly increases the risk of obesity and obesity-related outcomes [53]. Higher UPF consumption is inversely associated with vegetable, fruit, legume and seafood intake. Taken together, these findings show that the DP, more strongly than individual foods, impacts health outcomes.

As regards alcohol consumption, men exhibited a higher intake of calories derived from alcohol than women, which was in line with previously reported findings [54]. Whilst gender-specific differences were observed, in both cases, the energy intake derived from alcohol was not high, thereby demonstrating that it was not the main factor driving the energy imbalance between intake and expenditure. In fact, some studies have observed that PlwO exhibited lower rates of alcohol consumption as compared to the general population [55,56] showing that the higher the BMI, the lower the alcohol intake, probably in relation to overeating competing at brain-reward sites with alcohol [56,57]. This contrasts, however, with rodent studies in which some neuropeptides increased consumption of both high-fat foods and alcohol [58,59]. Noteworthily, frequency and quantity of consumption define hazardous drinking as well as its association with morbidity [60,61,62]. In this context, attention has been drawn to the relevance of drinking patterns and type of alcoholic beverages consumed, since some profiles of frequency and amount are associated with higher BMIs [63,64,65,66,67]. In our study, irrespective of gender, wine represented globally the main source of alcoholic beverages, with sugar-sweetened intake being above the optimal level in both genders. It was observed that beer intake was more frequent among people with a lower alcohol consumption frequency (<1 d/week) as compared to participants with a higher alcohol consumption frequency (5–7 d/week). In this respect, some authors suggest that in terms of contribution to body weight regulation, no relationship between alcohol intake and body weight exists [68,69]. However, via analyses of 5-year changes in alcohol consumption in relation to 5-year changes in WC and BMI, other authors have identified that the association of alcohol intake with measures of obesity is complex [70]. Thus, while in females intake of wine and distillates/mixed drinks exhibited contrasting associations with changes in WC and BMI, in males weekly alcohol consumption reduction with a special emphasis on stopping excessive intake could have beneficial effects on WC and BMI management. Of note, mean alcohol volume intake may mask the relationship between amount and frequency with health outcomes [63,71]. In the last years, an increase in alcohol consumption in adult females has been observed, thereby narrowing the gender gap [54]. Additionally, in spite of drinking less than men, women can reach higher circulating alcohol concentrations, thus experiencing more hazardous effects. However, except for hypertension risk, no gender-related effects of alcohol intake on coronary heart disease and stroke risk have been identified [62]. While the relationship is relatively linear between alcohol intake and hypertension for males, for women it does have a J shape, further evidencing the complexity of the gender-specific differences in alcohol intake. In this context, a higher adherence to a healthy beverage index reportedly minimizes the risk of metabolic alterations [72].

Suboptimal diets reportedly account for chronic disease development and more mortality globally than any other risks [3,18,73,74,75,76,77]. In the case of overweight and obesity, the focus of people pursuing weight loss has traditionally been on restricting single food items, which are blamed for leading to excess weight. In this respect, cultural beliefs frequently only blame beer [78], perpetuating conjectures and presumptions of unsubstantiated beliefs in the obesity domain [79]. Our study provides evidence that focusing on dietary and drinking patterns better captures the individual’s reality. Undoubtedly, in a complex activity like eating in which multiple homeostatic and hedonic factors are involved, focusing on a single item that can be blamed as the main culprit of excess weight represents a simplistic approach [80,81]. Data show that quantifying food consumption by both grams or binary variables yielded meaningful DPs, with each method providing different advantages; while weight accounts for food amount consumed, binary intake preferentially describes general food choices, which are potentially easier to be modified and therefore useful in public health settings [47]. In this context, governments should learn from their earlier policy failures [82] to prioritize policies that make minimal demands on individuals and have the potential for a population-wide reach so as to maximize their potential for equitable impacts. Moreover, policies should be proposed in ways that readily lead to implementation and evaluation.

Strengths of the study include the detailed patient phenotyping, the combination of tools aimed at achieving realistic nutritional information, and the experience of the research team in the field, which ensures reproducible study conditions. Our results provide clinically useful information relevant for therapeutic purposes. Given that foods are not consumed in isolation, identification of DPs based on actual eating behaviors yields more valuable interpretation and understanding of the diet-related causes of excess weight to help in the management of patients. Capturing the real DPs will help to develop a better understanding of the complexity of intake and to build trust in order to deliver effective evidence-based health interventions. Nonetheless, the present work also has some limitations. One potential limitation of the present study pertains to the generalizability to other populations, since the research was carried out in the homogeneous sample population attending our Department for weight loss, thereby overturning the potential effect of confounding factors such as race, ethnicity, educational level, cultural beliefs, lifestyle factors and socioeconomic status, among others. However, this aspect may be also viewed as a strength, since the sample homogeneity ensures the lack of potential confounders. The findings would need to be confirmed in other populations in order to determine potential ethnic or race-specific differences as regards DPs, body composition and associations with the cardiometabolic risk factors.

## 5. Conclusions

The focus on consumption of single nutrients does not resemble real-world intake behaviors. As hypothesized, capturing the DP provides more clinically relevant and useful information. The obtained data can provide useful information for strategic approaches to improve the effectiveness of obesity policies. Healthcare practitioners should be encouraged to offer dietary policies that focus on the promotion of diet components for which non-optimal intakes are evident, rather than only targeting individual or specific food items. The evidence provided in the present study can help to broaden and implement policy decision making for obesity management with DP modification, yielding more long-term benefits in terms of sustainability and feasibility of interventions.

## Figures and Tables

**Table 1:** Anthropometric and clinical characteristics of the study participants.

Variables	Mean ± SEM (*n* = 200)
Sex (males/females)	56/144
Age (years)	38 ± 12
BMI (kg/m^2^)	32.2 ± 3.5
Body fat (%)	40.0 ± 4.3
Visceral adiposity (a.u.)	13.9 ± 3.7
WC (cm)	97 ± 15
Systolic BP (mm Hg)	131 ± 16
Diastolic BP (mm Hg)	85 ± 10
Glucose (mg/dL)	98 ± 17
Insulin (μU/mL)	11.9 ± 3.5
HOMA-IR	2.87 ± 0.91
QUICKI	0.330 ±0.044
Triglycerides (mg/dL)	103 ± 10
Total cholesterol (mg/dL)	208 ± 22
HDL-cholesterol (mg/dL)	53 ± 8
LDL-cholesterol (mg/dL)	130 ± 9
Free fatty acids (mEq/L)	0.5 ± 0.1
Creatinine (mg/dL)	0.80 ± 0.11
Uric acid (mg/dL)	4.5 ± 0.2
Urea (mg/dL)	12.4 ± 1.3
ALT (UI/L)	19 ± 7
AST (UI/L)	25 ± 8
AP (UI/L)	66 ± 9
GGT (UI/L)	28 ± 9
Leptin (ng/mL)	36.5 ± 6.1
Adiponectin (μg/mL)	6.7 ± 2.1
Osteopontin (ng/mL)	68.8 ± 9.4
Lipocalin 2 (ng/mL)	70.2 ± 3.6
Tenascin C (ng/mL)	63.1 ± 3.7
YKL-40 (ng/mL)	36.0 ± 4.4
TNF-α (pg/mL)	6.37 ± 3.52
MCP-1 (pg/mL)	486 ± 200
IL-1β (pg/mL)	5.15 ± 1.02
IL-4 (ng/mL)	3.41 ± 1.18
IL-6 (pg/mL)	4.42 ± 0.79
IL-8 (pg/mL)	4.31 ± 1.25
IL-10 (pg/mL)	6.52 ± 2.38
IL-32 (pg/L)	10.5 ± 2.3
CRP (mg/L)	5.3 ± 2.7
vW Factor Ag (%)	133 ± 28
Homocysteine (μmol/L)	8.72 ± 0.79
Fibrinogen (mg/dL)	286 ± 25

BMI, body mass index; a.u., arbitrary units; WC, waist circumference; BP, blood pressure; HOMA-IR, homeostasis model assessment—insulin resistance; QUICKI, quantitative insulin sensitivity check index; ALT, alanine aminotransferase; AST, aspartate aminotransferase; AP, alkaline phosphatase; Gamma-GT, gamma-glutamil transferase; YKL-40, chitinase 3-like protein-1; TNF-α, tumor necrosis factor alpha; MCP-1, monocyte chemotaxis protein 1; IL, interleukin; CRP, C reactive protein; vW factor Ag, von Willebrand factor antigen.

**Table 2:** Total caloric intake derived from alcohol consumption.

Energy	Females			Males			Stat. Sign.
	Mean	Minimum	Maximum	Mean	Minimum	Maximum	
**Total energy intake (TEI) (kcal/d)**	2252	1219	3562	2494	1915	4158	*
**EI from alcohol (kcal/d)**	73	0	430	96	0	482	*
**EI from alc. (% of total)**	2.9	0	12.1	4.5	0	15.4	*

EI from alc, energy intake from alcohol. Data are presented as mean followed by minimum and maximum values in brackets. Stat. sign., statistical significance; * *p* < 0.05 assessed by Chi-square (χ^2^) analysis.

**Table 3:** Proportion of total alcohol intake according to type of beverage and frequency of consumption.

Type of Beverage	Females Frequency (Drinks/Week)			Males Frequency (Drinks/Week)			Stat. Sign.
	(%)	<1	5–7	(%)	<1	5–7	
**Beer**	18.8	23.7	13.8 #	41.0	48.0 §	38.6	*
**Wine**	69.3 §	52.6 §	79.4 §	46.3	31.6	51.0 §	*
**Distillates**	11.9	23.7	6.9 #	12.7 §	20.3 §	10.5 #	ns

Distillates, liquors and distilled spirits. Data are presented as mean percentage values. Stat. sign., statistical significance; Chi-square (χ^2^) analysis was applied to determine differences in gender and categorical variables; ns, non-significant for gender; *p* < 0.05 assessed by χ^2^ for * gender, § type of beverage and # frequency of consumption.

**Table 4:** Global proportion of participants with a low intake of foods in relation to frequency of alcoholic drinks per week.

Diet Poor in	Females Freq. (Drinks/Week)			Males Freq. (Drinks/Week)			Stat. Sign.
	(%)	<1	5–7	(%)	<1	5–7	
**Fib. and cer.**	71.4 §	70.2	79.5	74.8 §	76.3	65.8	ns
**Vegetables**	66.8 §	61.3	72.0	71.0 §	74.8	53.7 #	ns
**Salads**	55.2 §	57.5	53.1	69.4 §	68.6	63.1	*
**Legumes**	87.3 §	85.4	89.6	85.3 §	88.7	83.6	ns
**Fruits**	20.0	15.2	24.1 #	39.2	39.0	38.5	*
**Fish**	60.2	63.6	57.8	68.1	67.8	60.0	ns

Fib. and cer., Fiber and cereals; Freq., Frequency. Data are presented as mean percentage values. Stat. sign., statistical significance; Chi-square (χ^2^) analysis was applied to determine differences in gender and categorical variables; ns, non-significant for gender; *p* < 0.05 assessed by χ^2^ for * gender, § type of beverage and # frequency of consumption.

**Table 5:** Global proportion of participants with elevated intake of foods in relation to frequency of alcoholic drinks per week.

Diet High in	Females Fre. (Drinks/Week)			Males Freq. (Drinks/Week)			Stat. Sign.
	(%)	<1	5–7	(%)	<1	5–7	
**Sweets**	93.1 §	92.4	91.7	79.6 §	80.3	82.1	*
**Solid fats**	76.5	75.3	77.2	77.8	79.0	75.4	ns
**Ch./cold mts**	49.9 §	50.6 §	48.8 §	83.4 §	81.2	84.7	*
**Sug.-sw. bev.**	80.7 §	80.2	81.0	81.2 §	80.3	83.6	ns

Solid fats, butter and margarine; Ch./cold mts, cheese/cold meats; Sug.-sw. bev., sugar-sweetened beverages. Data are presented as mean percentage values. Stat. sign., statistical significance; Chi-square (χ^2^) analysis was applied to determine differences in gender and categorical variables; ns, non-significant for gender; *p* < 0.05 assessed by χ^2^ for * gender, § type of beverage.

## Data Availability

The data presented in this study are available upon reasonable request from the corresponding author. The data are not publicly available due to privacy restrictions.

## Abbreviations

ALT: alanine aminotransferase; AST: aspartate aminotransferase; AP: alkaline phosphatase; BMI: body mass index; BP: blood pressure; CRP: C reactive protein; CV: coefficient of variation CVD: cardiovascular diseases; DP: dietary pattern; ELISA: enzyme-linked immunosorbent assay; FFQs: food frequency questionnaires; Gamma-GT: gamma-glutamil transferase; HOMA-IR: homeostasis model assessment—insulin resistance; IL: interleukin; ISAK: International Society for the Advancement of Kinanthropometry; LCN-2: lipocalin-2; MCP-1: monocyte chemotaxis protein 1; OPN: osteopontin; PAL: physical activity level; PlwO: people living with obesity; QUICKI: quantitative insulin sensitivity check index; REE: resting energy expenditure; RRR: reduced-rank regression; TEE: total energy expenditure; TEF: thermic effect of food; TNC: tenascin C; T2D: type 2 diabetes; TNF-α: tumor necrosis factor alpha; YKL-40: chitinase 3-like protein-1; vW factor Ag, von Willebrand factor antigen; WC: waist circumference; WHO: World Health Organization.
